# Respirometric Study of Optical Brighteners in Textile Wastewater

**DOI:** 10.3390/ma12050785

**Published:** 2019-03-07

**Authors:** Héctor Salas, Carmen Gutiérrez-Bouzán, Víctor López-Grimau, Mercedes Vilaseca

**Affiliations:** 1 Institute of Textile Research and Industrial Cooperation of Terrassa (INTEXTER), Universitat Politècnica de Catalunya—BarcelonaTech, Colom 15, 08222 Terrassa, Spain; m.carmen.gutierrez@upc.edu (C.G.-B.); victor.lopez-grimau@upc.edu (V.L.-G.); m.merce.vilaseca@upc.edu (M.V.); 2 Department of Project and Construction Engineering, Terrassa School of Industrial, Aerospace and Audiovisual Engineering (ESEIAAT), Universitat Politècnica de Catalunya—BarcelonaTech, Colom 11, 08222 Terrassa, Spain

**Keywords:** optical brighteners, bacterial activity, respirometry, biodegradability, textile wastewater

## Abstract

Optical brighteners (OBs) are colorless fluorescent dyes, widely used in industry to improve whiteness in materials. Nearly 80% of all OBs in the market are derivatives of stilbene. They absorb the near-ultraviolet light and re-emit most of it in the blue range as visible fluorescence. OBs are commonly applied on textiles, detergents, paper and plastic products, among others. OBs have a low degradation ratio. In biological plants, they can only be partially removed by adsorption into the sludge and a tertiary treatment could be required to fully remove them. Part of them may persist and can be found in river and lake waters. The current work aims to evaluate the effect of the OBs in the bacterial activity of biological wastewater treatment plants. The influence of two commercial OBs (Goldblanc BHA and Leucophor PC) on respiration rate was monitored by means of a semi-continuous electrolytic respirometer, in order to obtain information related to the growth of the biomass and the degradation of the substrate. Their acute toxicity was also determined. It was concluded that the OB effect on bacteria population is variable depending on its chemical structure. Unlike the former, the Leucophor-PC brightener had an impact on the respirometric rate.

## 1. Introduction

White color in textile products is commonly associated with purity and cleanliness. For this reason, chemical bleaching processes are applied to improve the commercial value of textile goods. During the chemical bleaching, impurities are destroyed or decolored by oxidation or reduction. However, in chemically bleached fabrics, a slight yellow color still remains, which may diminish their aesthetic appeal [1]. In order to overcome this problem Optical brighteners (OBs) are used to reach a bright white in white textiles, as they are effective to avoid yellowish shade in white products and to give a higher sensation of white.

Optical brighteners (OBs) are colorless fluorescent dyes. When they are exposed to solar light, they absorb the near-ultraviolet light and re-emit most of it in the blue range as fluorescence visible under UV light.

In addition to textile, OBs are widely used in different industries to improve whiteness in many materials. OBs are also used in some colored materials to make them seem brighter [1,2,3]. They are commonly used in detergents, paper (including toilet paper) and plastic products. OBs also are used in the photographic industry, feather, leather, furs, paints, fats, gelatin, printing inks, adhesives, circuit boards and coating for golf balls, appliances, etc. [3,4].

With respect to the chemical structure of OBs, they can be derivatives from stilbene, benzoxazole, coumarin, pyrazolines, etc. This study focused on OBs derived from stilbene, because these are nearly 80% of all OBs in the market. Stilbene OBs structure are characterized by a central ethene double bond with a phenyl group on both carbon atoms [2].

Textile finishing and laundry sectors are some of the greatest generators of wastewater containing OBs. The majority of these OBs are used on bleaching of cotton fabrics in which have low fatness to washing. Moreover, they are also present in domestic wastewaters. The bleaching residual effluents can be more or less harmful to the environment, depending on the chemical structure of the selected OB and on the amount of salts used to improve OBs sorption during the whitening process [2,5,6].

Regarding the toxicity of the OBs, contradictory information has been found in the literature. However, it has been reported that some OBs are potentially carcinogenic and mutagenic and, in high concentrations, can have a negative impact on aquatic organisms [7,8].

OBs have a very low degradation ratio. In the environment, OBs can be partially degraded and isomerized by sunlight, but part of them may persist and can be found in river and lake waters. In the biological treatment plants, OBs can be only partially removed by adsorption into the sludge and a tertiary treatment could be required to fully remove them. When OBs are not properly removed they can be found in the aquatic sediments, so that the animal and plant life could be affected [6,9,10]. The OBs may also affect to the microorganisms in the biological systems.

The current work aims to evaluate the effect of the OBs in the bacterial activity of biological wastewater treatment plants. In order to obtain information related to the growth of the biomass and the degradation of the substrate, the respiration rate (RR) of *Vibrio ficheri* bacteria was monitored by means of a semi-continuous mode electrolytic respirometer.

## 2. Materials and Methods

**Figure 1:**
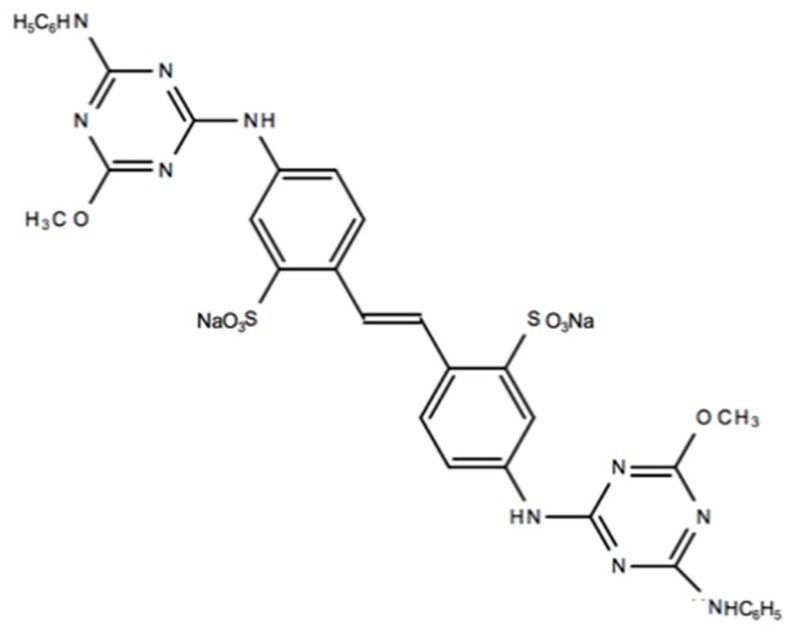
L-PC chemical structure.

Two liquid OBs, derivatives from stilbene, were selected in this study:

- Fluorescent Brightener 134, commercial name Leucophor PC (Clariant Iberica, Sant Joan Despí, Spain) and identified in this document as “L-PC”. It is a diaminostilbene-disulphonic acid derivate product with CAS Number 3426-43-5. It can be found in the Colour Index as Fluorescent Brightener N/A 134. Its chemical structure is presented in Figure 1.
- A commercial optical brightener provided by Golden Technology Inc. (Golden Technology, São Paulo, Brazil), named Goldblanc BHA and referred in this document as “BHA”. It is a derivative of 4,4′-diaminostilbene-2,2′-disulfonic acid (DSD acid). This OB is currently used in the textile industry. It was not possible to obtain more information beyond the data provided by the manufacturer. For this product, the full chemical composition and structure has not been published because it is confidential information of the provider.

The toxicity data indicates that these OBs are irritant substances. L-PC toxicity data was obtained by CAS number internet search and for BHA from the safety data sheet. Both are categorized as non-hazardous substances; however, they are harmful to aquatic life [11].

To conduct the experiments, solutions were prepared as follows: OBs were homogenized in distilled water by a magnetic stirring at room temperature (25 °C). Concentrations from 150 to 400 mg/L were tested. OB solutions were adjusted to pH 7 with NaOH and HCl 0.1 N. Analytical methods performed are listed below.

The density and dry matter of OBs were determined by gravimetric method at 25 °C. The density was evaluated based on mass and volume of each OB at room temperature (25 °C) as follows: 10 mL of OB was weighted on an Ohaus Explorer analytical balance (Ohaus, Parsippany, NJ, USA, accuracy of 0.0001 g). Density outcome in laboratory was 1.21 g/cm^3^ on L-PC and 1.13 g/cm^3^ on BHA. 

The content of water on each OB was evaluated. The samples were dried in oven for 72 h at 60 °C or until constant weight. The BHA had 60% w/w of water and L-PC had 25% w/w of water. As L-PC was more concentrated than BHA, the last was easily homogenized.

### 2.1. Organic Load of OBs

The organic load characterization of OBs in solution was evaluated based on dry weigh. Total organic carbon (TOC), chemical oxygen demand (COD) and biochemical oxygen demand (BOD) were analyzed.

TOC was determined with a Shimadzu TOC analyzer (based on EN 1484:1997 standard) [12]. With this analysis, the amount of organic carbon in solution was measured, more specifically, the non-purgeable organic carbon.

COD was evaluated by a dichromate method (based on ISO 6060:1989 standard) [13]. This test analyzes the amount of oxygen required to oxidize entirely the organic matter in solutions.

BOD was determined after a five-day period (BOD_5_) at 20 °C ± 1 °C [14]. This test measured the oxygen consumed by the microorganisms for the degradation of the organic material. The initial bacterial inoculum was obtained from a soil suspension. It was assumed that the microorganisms in the soils are the similar to those contained in the biological sludge [15].

### 2.2. FTIR Analysis

Infrared spectra were recorded in order to identify the chemical structure of the samples using Fourier-transform infrared (FTIR) spectroscopy. OBs samples were dried at 40 °C for 72 h in glass petri dishes and were analyzed with Thermo Scientific Nicolet 6700 FTIR (Thermo Fisher Scientific, Waltham, MA, USA) with attenuated total reflectance (ATR) accessory. Analysis was performed in the wavelength range between 4000 and 400 cm^−1^, with a resolution of 4 cm^−1^ and an average of 32 spectral scans accumulated. IR spectra were cross-checked with different databases. It was possible to identify some structural differences of the two OBs studied based on the obtained spectrum.

### 2.3. Toxicity

Toxicity evaluation of chemicals is of major importance to avoid negative impact on living organisms and environmental pollution.

Acute toxicity was tested detecting the inhibition of the luminescence of the bacteria *Vibrio fischeri*, as indicated in the ISO 11348-3:2007 Standard [16]. Microtox M500 equipment (Microbics Corporation, Carlsbad, CA, USA) was used. In the test, OB solutions are in contact with the bacteria for 15 min. Toxicity test results can indicate if certain OB concentration affects up to 50% of the organism population [17,18]. This toxicity test is widely used because it is a fast and effective way to detect acute toxicity. Toxicity test with *Vibrio fischeri* was selected because it can be applied to multiple substances, including wastewaters organic and inorganic compounds, among others [19]. In particular, this toxicity test has been used successfully to evaluate dyestuffs and auxiliaries from textile processes [17,20,21].

### 2.4. Respirometry

Respirometry is a fundamental tool for the control of biochemical processes and usually is employed to design biological treatment plants. This respirometric test allows monitoring of the biological oxygen consumption rate. Respirometry can provide information about biodegradability of the OBs. It is useful to determine whether there are affectations in the biological system [22]. Microbial metabolism was monitored by means of an electrolytic respirometer (BI-2000, Bioscience Inc., Sherwood, OR, USA). It was mainly constituted of one module with multiple reactors, where the sample with the inoculum and the necessary nutrients (the same as those used in the BOD test) were placed. Each reactor had a trap with alkali (45% KOH solution) that absorbs the CO_2_ produced by microorganisms in the decomposition of organic matter. Additionally, an electrolytic cell was attached, loaded with H_2_SO_4_ 1 N. The experiments were conducted with continuous magnetic stirring in a thermostatic bath at 20 °C.

The inoculum used in the respirometric tests was a mixture of three bacterial aggregates in equal parts (MICROCAT SX, XP and HX, Bioscience Inc.) at 100 mg/L.

Three different MICROCAT bioformula, were used in equal proportion (33.33% of the inoculum). This mixed MICROCAT inoculum was used instead of activated sludge in order to provide more reproducible and accurate information about the biodegradability of wastewaters [23]. This microorganism mixture could be more sensitive to toxic or inhibitory substances than an adapted sludge inoculum [24].

Respirometry was monitored up to 180 h. Solutions with different concentration of OBs were tested, from 150 to 400 mg/L, which is the higher concentration recommended in industrial whitening recipes at a liquor ratio of 1:10. The effect of sodium sulfate was also evaluated, as it is used as electrolyte in the textile industry to improve whitening processes. In this case, it was used at 5 g/L in BHA solutions samples [25].

## 3. Results and Discussion

### 3.1. Organic Load Results

The results of organic load of OBs in solution are presented in Table 1. Biodegradability of OBs was evaluated by the BOD_5_/COD ratio. This value is considered by some authors as the biodegradability index [26], in this case it was measured for 5 days. Commonly, very biodegradable wastewaters have BOD/COD ratios greater than 0.5. Values from 0.5 to 0.2 are related to medium or slowly biodegradable waters and values lower than 0.2 refer to very poor or not biodegradable waters [27].

In this case, L-PC had slightly higher TOC (0.39 mg C/mg) than BHA (0.33 mg C/mg), whereas COD was significantly higher on L-PC (1.28 mg O_2_/mg) than BHA (0.77 mg O_2_/mg). Thus, the COD of L-PC was almost twice than BHA, despite the TOC being slightly similar.

The TOC values are a direct measure of the organic load but COD is an indirect measure as it indicates the oxygen required to chemically oxidize these compounds. Therefore, it can be concluded that BHA is oxidized more easily than L-PC.

BOD_5_ was lower in L-PC (0.20 mg O_2_/mg) than BHA (0.29 mg O_2_/mg). Based on the results, it can be stated that both OBs are not very biodegradable, as their BOD_5_/COD ratio was lower than 0.5. In fact, it should be pointed that BHA has low biodegradability (0.37) and L-PC was very poorly biodegradable (0.16).

### 3.2. FTIR Analysis Results

**Figure 2:**
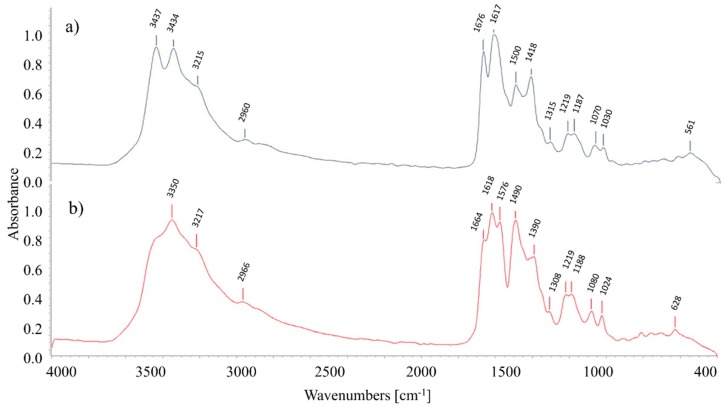
(**a**) FTIR spectrum of BHA; (**b**) FTIR spectrum of L-PC.

FTIR spectra were recorded in order to complement the OBs physicochemical characterization. In Figure 2 are shown the spectra of BHA (Figure 2a) and L-PC (Figure 2b). Nevertheless, it was not possible to obtain the exact chemical identification of BHA using different databases. Similarities and differences between both OBs spectra were analyzed below.

When comparing both spectra, a high similarity between them is appreciated. In the range 1600–1450 cm^−1^, the L-PC has slightly higher intensity in two bands, which may indicate a greater amount of aromatic and aliphatic carbon bonds. This would confirm the previous results about the slightly higher organic carbon load of L-PC with respect to BHA.

Moreover, in this wavelength range, bands at 1250–1140 cm^−1^ and 1070–1030 cm^−1^, observed in both spectra can be attributed to S=O bonds [28].

In the wavelength range from 3600–3200 cm^−1^, a double peak in BHA spectrum may be correlated with a primary amine (–NH_2_), while in a single peak in L-PC spectrum can be attributed to a secondary amine (–NH). Another difference is that BHA has a more marked waveband at 1676 cm^−1^, which means a higher presence of C=O bonds than in L-PC. This indicates that the BHA molecule is more oxidized than L-PC, which is in accordance with the COD results.

FTIR spectra of BHA and L-PC were compared with FTIR data of other triazine-stilbene OBs published in literature related to the synthesis and the structural analysis of OBs derivatives of DSD acid-triazine. FTIR spectra of these OBs showed two peaks corresponding to triazine-ring skeleton vibrations [29,30,31,32]. In the case of BHA these peaks were at 1418 and 1500 cm^−1^ and in the case of L-PC at 1390 and 1490 cm^−1^.

In general, the study of FTIR spectra does not allow to elucidate the complete chemical structure of BHA and L-PC. However, the information provided by the manufacturer could be corroborated.

### 3.3. Toxicity Results

L-PC and BHA were tested at 1 g/L, the concentration generally applied in textile whitening processes (liquor ratio 1:10). Both OBs were “non-toxic” or “non-luminescence inhibitor for *Vibrio fischeri*” at this concentration. Then, the toxicity result is expressed as: 15 min EC50 > 1000 mg/L. Considering only these results, it is suggested that these substances did not harm the microorganism directly.

Despite these toxicity results, respirometric test results must be carried out because rapid bioassays (as *Vibrio fischeri*) did not show alterations in bacteria over a prolonged period.

### 3.4. Respirometry Results

Respirometry was carried out with inoculum obtained from specialized wastewater treatment bacteria, in contrast to the BOD test that was performed with non-specialized soil bacteria. These specialized bacterial aggregates are highly resilient. It was expected that they had high biological activity in the respirometric test, since they would adapt better in the aqueous medium with OB [22].

**Figure 3:**
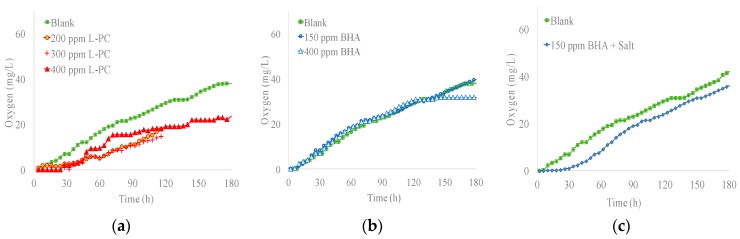
(**a**) Respirometry of L-PC; (**b**) Respirometry of BHA; (**c**) Respirometry of BHA with salt.

Respirometric rate (RR) results of both OBs are shown in Figure 3. RR of L-PC solutions at 200, 300 and 400 mg/L are showed in Figure 3a. BHA at 150 mg/L and 400 mg/L concentrations are presented in Figure 3b. The influence on the RR of salt, sodium sulfate, in BHA samples was also monitored for 180 h (Figure 3c).

L-PC was less biodegradable than the blank along the whole test (Figure 3a). As is known, a drop or absence of respiration rate is indicative of a possible inhibition or toxicity [22]. L-PC solutions at 200 mg/L or higher can decrease the biological activity. In the blank sample, without OBs, bacterium adaptation time was minimal, around 8 h. In the other samples, BO generated harmful conditions and stress for these microorganisms. Therefore, in the first 30 h, correlated to the adaptation period of microorganisms, the RR was minimal. After this period there was a regular increase in bacterial activity. After 180 h, the RR in OB samples was around 35% lower than the blank. It was also observed that low BHA concentrations affected microbial activity.

RR in BHA remained close to the blank at least for the first 150 h (Figure 3b). These solutions appeared not to affect the biological activity. When the OBs remained in the reactor, no inhibitory effects were observed for BHA solutions.

As stated before, BHA at 150 mg/L was not adverse for the biological activity. However, in BHA sample with 5 g/L of salt the RR performance was lower than blank (Figure 3c) and lower than BHA samples (Figure 3b). In this case, the alteration of the biological activity was not caused by the OB product, but rather by the presence of electrolyte. RR was minimal during the first 30 h in salt samples. After 180 h the RR was 5% lower than the blank. It is known that moderate or high salinities can produce inhibitory or toxic effects on bacteria not adapted to high salinity [33]. These conditions can cause loss of activity of cells and could even affect other biochemical properties of the activated sludge.

The effect of salts in biological activity was variable for each OB. This can be attributed to the presence of different functional groups on the benzene rings on the stilbene for each compound [34]. Therefore, the effect of OBs on biological activity can be more or less harmful depending on the type of stilbene derivate substances.

The results suggest that BHA was less harmful to microbiological activity than L-PC. BHA is a newer commercial product than L-PC. However, this does not guarantee to have low environmental impact. It is necessary to keep in mind that L-PC is a more concentrated OB than BHA. Therefore, concentrated OBs may have greater impact on biological treatments and on the environment (if they are dosed at the same ratio). Additionally, diluted products are usually easier to handle and may be cheaper than the concentrated ones.

## 4. Conclusions

Physicochemical properties, toxicity and respirometric data of two different OBs, stilbene derivatives, were studied.

L-PC proved to be more resistant to chemical oxidation. This stability is an important factor during the choice of the brightener to be used in the textile fibers. It is expected that OBs are resistant to attack from oxidants products that can be found in commercial detergents. However, more stable OBs can be also more difficult to be removed from the environment.

On the other hand, it should be pointed that BHA is not a risky product for the microorganisms of biological systems. However, it is also resistant to biodegradation. Therefore, if this product reaches the environment, it could accumulate mainly in the aquatic sediments.

With respect to the toxicity, the fast acute toxicity tests can provide important information to determine if these substances are harmful to microorganisms in water. However, this data are not sufficient to evaluate the OB effect on bacteria in biological wastewater treatments. To evaluate if OBs or other compounds that can inhibit bacterial growth, it is necessary to perform longer acute toxicity tests or chronic toxicity tests. However, the respirometry provides important and relevant information related to bacteria growth inhibition.

The respirometric tests allow to monitor continuously the performance of the bacterial activity and its alteration due to the effect of OB. This study showed that some OBs may decrease the respirometric rate of the microorganisms, being its effect variable for each OB composition. Then, it can be concluded that the optical whitening wastewater effluents can be harmful to the biological treatment systems because of the residual OB used in these whitening textile processes. Additionally, the residual electrolytes employed in these processes can alter the bacterial activity.

## Figures and Tables

**Table 1:** Organic load obtained per dry weight of Optical Brighteners (OBs).

OB	COD (mg O_2_/mg)	BOD_5_ (mg O_2_/mg)	TOC (mg C/mg)	BOD_5_/COD Ratio
L-PC	1.28	0.20	0.39	0.16
BHA	0.77	0.29	0.33	0.37
